# The clinical burden of microscopically patent and sub-microscopic *P. falciparum* and *P. vivax* malaria in pregnancy in Indonesia

**DOI:** 10.1186/1475-2875-13-S1-P2

**Published:** 2014-09-22

**Authors:** Rukhsana Ahmed, Puji PB Asih, Rintis Noviyanti, Ismail EP Rozy, Leily Triyanti, Rita Marleta, Feiko ter Kuile, Din Syafruddin

**Affiliations:** 1 Liverpool School of Tropical Medicine, Pembroke Place, Liverpool, UK; 2 Eijkman Institute for Molecular Biology, Jl Diponegoro 69, Jakarta, Indonesia; 3 National Institute of Health Research and Development, Ministry of Health, Jakarta, Indonesia

## Background

Malaria in pregnancy poses a major public health problem in Indonesia with an estimated 6 million pregnancies at risk of *P. falciparum* or *P. vivax* malaria annually. The association between microscopically patent and PCR positive sub-microscopic malaria with maternal anaemia and low birth weight babies was assessed in South-west Sumba and Jayapura district, Papua, Indonesia.

## Materials and methods

Cross sectional surveys were conducted between June 2008-May 2009 in antenatal women and at delivery including home deliveries in SW Sumba (low-moderate transmission) and Jayapura (high transmission). Maternal and placental blood samples were obtained at antenatal attendance and at delivery for haemoglobin measurement, smear microscopy and PCR.

## Results

A total of 4230 women (2598 attending antenatal care and 1632 at delivery) were enrolled. The prevalence of maternal parasitaemia detected by microscopy was 7.0% [(180/2598) (mono-infection with *P. falciparum*: 58.3%, with *P. vivax*: 20.6% and 0.5% *P. ovale* and 5.6% mixed infections (*P. falciparum* + *P. vivax*), and this was 12.3% by PCR (*P. falciparum* 58.4%, *P. vivax* 31.5%, mixed (*P. falciparum* + *P. vivax*): 10.1%) and 10.7% in Sumba and 14.2% in Jayapura, respectively. Sub-microscopic parasitaemia (microscopy negative, PCR positive) was detected in 8.3% women. With patent infections only *P. falciparum* was associated with moderate-severe anaemia (Hb ≤9 g/dL); [RR 2.2 (95% CI 1.7-2.9) *P. vivax* RR 0.5 (0.3-0.9)] whereas with sub-microscopic infections both species were associated with increased risk of moderate to severe anaemia [*P. falciparum*: RR 2.1 (95% CI 1.6-2.8) and *P. vivax*: RR 1.9 (95% CI 1.3-2.7)]. The prevalence of placental malaria was 4.8% (72/1632) by PCR and 3.3% sub-microscopically; this was 2.4% in primigravidae, 3.1% in secundi and 1.7% in multigravidae (≥3 pregnancies). The mean difference in birth weight in women with patent placental *P. falciparum* infection compared with uninfected women was 101 g (95% CI 70-273 g) with RR 2.1 (95% CI 1.2-3.0) for low birth weight. The reduction in birth weight with sub-microscopic infection was 70 g (63-204 g) with low birth weight RR 1.7 (95% CI 1.0-2.5). Corresponding figures for *P. vivax* were: patent placental infections: 252 g (57-560 g), low birth weight RR 4.7 (95% CI 2.2-67); and for sub-microscopic infections the mean difference in birth weight was 146 (70-364 g); low birth weight RR 2.9 (95% CI 1.6-3.4).

## Conclusions

Sub-microscopic infections are common in eastern Indonesia and are associated with maternal anaemia and marked reductions in birth weight. Strategies that prevent sub-microscopic infections such as intermittent preventing therapy or that which results in their early detection and treatment, such as intermittent screening strategies should now be explored in South-east Asia.

